# How collaborative, interpersonal, and disaster-responsive tendencies work together in non-face-to-face environments: lessons from prolonged pandemic experiences

**DOI:** 10.3389/fpsyg.2024.1414235

**Published:** 2024-08-26

**Authors:** Soyoung Kim, Simon Andrew, Richard C. Feiock, Christopher Stream

**Affiliations:** ^1^College of Liberal Arts, Seoul National University of Science and Technology, Seoul, Republic of Korea; ^2^College of Health and Public Service, University of North Texas, Denton, TX, United States; ^3^Local Governance Research LLC, Tallahassee, FL, United States; ^4^Greenspun College of Urban Affairs, University of Nevada, Las Vegas, Las Vegas, NV, United States

**Keywords:** prolonged disaster, non face-to-face environment, collaborative characteristics, interpersonal reactivity, disaster response, canonical correlation, mediation and moderation

## Abstract

This study investigates the dynamics of collaborative characteristics, interpersonal reactivity, and disaster situation responses in non-face-to-face settings, a response mechanism increasingly relevant in the wake of prolonged pandemics. By examining a group of 123 university students engaged in a seven-week non-face-to-face collaborative project, the research identifies relational patterns between collaborative traits such as regulation and efficacy, interpersonal empathy, and responses to disaster situations. The research methodology employs a sophisticated analytical framework comprising factor and canonical correlation analyses to identify how empathy and collaborative efficacy significantly related with disaster response in online collaborations. The mediation and moderation models analyzed confirm mutual mediation effects of collaborative regulation and interpersonal reactivity on situational empathy without significant moderation effects. This suggests there were direct causal relationships of collaborative regulation, collaborative efficacy, interpersonal reactivity on situational empathy. The findings underscore the pivotal role of empathy in collaboration during disasters offering a nuanced understanding of the social and psychological underpinnings that enable collective responses to crises in environments lacking physical interaction and illuminating the critical role of collaborative and interpersonal skills in such settings.

## 1 Introduction

The recent pandemic forcefully demonstrated that prolonged disasters can disrupt natural and human interactions to produce a cascade of social problems. However, this experience also provides collective experience in maintaining interactions and sustaining social activities, albeit in limited online ways (Chauhan et al., 2024). The remarkable technological advances, including the widespread adoption of video conferencing tools and the integration of collaborative platforms, have significantly strengthened the online environment during the pandemic (Camilleri and Camilleri, 2021). As a result, many scholars predict that since humanity has become more accustomed to indirect interactions through non-face-to-face methods, people will work and learn together in online environments more frequently in the future (Bashir et al., 2021; Heo and Lim, 2021; Zhang et al., 2022).

In an era where unexpected disasters are becoming more frequent, diverse, and complicated, collaboration and social skills based on people-to-people relationships will be emphasized even more urgently to overcome crises (Jewett et al., 2021). A disaster has been defined as an event that occurs unexpectedly and severely impairs the functioning of a community or society (Norris et al., 2008). Disrupted functioning means that human, material, and economic losses have occurred that are beyond the capacity of a society to bear, and these consequences can be natural or man-made, often leading to socioeconomic repercussions (Bankoff et al., 2004; van Bavel et al., 2020). In our times, disasters inevitably become long-term problems.

The ability of a community to respond is an important determinant of how a disaster unfolds. The history of disasters reveals that societies and communities have responded differently to similar crises, with different outcomes (Zamoum and Gorpe, 2018; van Bavel et al., 2020). Disasters are more overwhelming for groups that are vulnerable to hazards, and their impact is intensified when collective capacities fail to reduce the force of hazards (Mutter, 2015; Drury et al., 2019). It is possible to say that calamities are more destructive when communities lack the capacity to respond to them collectively.

Social competencies such as empathy and collaboration play an important role in a community’s effective response to a crisis. Disaster studies conclude that the altruistic and collaborative behaviors that emerge in crisis situations are responsible for effectively overcoming the situation, saving lives, and reducing psychological distress (Cole et al., 2011; Drury et al., 2019). The problem is that social capacities to overcome crises, such as empathy, social responsibility, altruism, collaboration, and teamwork, are formed and expressed only through social interactions (Adolphs, 2009; Graziano, 2013). At a time when non-face-to-face activities have increased and are expected to increase further, the question naturally arises as to whether these social competencies can be fostered, worked, and influenced in online environments that lack direct interactions to facilitate collaborative activities.

Recent neuroscience explains that social responses, including empathy, are expressed, learned, and stored in memory through the mechanisms of mirror neurons, the role of the amygdala and hippocampus, and the function of the frontal lobe to look at each other, understand situations, and accept the emotions of others (Marsh, 2016). Interpersonal emotional responses such as empathy are central to altruistic collaborative behaviors (Muller et al., 2014). The amygdala and hippocampus, in particular, are responsible for the expression of emotions such as empathy and fear, and for triggering behavior (Pfaff, 2015). It is worth exploring whether the social responses that occur when we encounter others can also influence our collaborative behavior in online settings.

Neuroscience explains that these social characteristics as both genetic predispositions that individuals are born with and the result of acquired learning that is reshaped through collaborative experiences (Wagner and Rush, 2000; Zaki and Ochsner, 2012; Marsh, 2016). Empathy and altruism, which originate in the brain’s amygdala, are responses to the emotion of fear in others (Keysers, 2011; Marsh, 2016). A low level of reactivity to another person’s fear leads to selfish behavior, while a high level of reactivity causes us to feel distressed at the mere sight of another person’s suffering (Marsh et al., 2014; van Dongen, 2020). These brain operations are powerful enough to help others; therefore, groups survive and engage in collaborative behavior during disasters.

It is not merely the experience of a disaster that determines the extent of empathy and collaboration among individuals and groups, but rather how these experiences are interpreted (Vardy and Atkinson, 2019). The aftermath of a disaster can lead people to become either more altruistic or more selfish, depending on their interpretation and processing of the experience (Li et al., 2013; Drury et al., 2019). Vardy and Atkinson (2019) argue that witnessing others’ suffering tends to reinforce collaborative and altruistic tendencies. However, experiencing economic hardship after a disaster may lead to more selfish behavior in similar situations. This suggests that our perceptions during prolonged disasters, such as pandemics, may shape our future social responses and collaboration.

Empathy is the ability to understand and share the feelings and behaviors of others, and it plays a crucial role in enhancing social cohesion (Keysers, 2011; Zaki and Ochsner, 2012; van Dongen, 2020). Despite various interpretations of empathy, it is widely accepted as a multifaceted concept comprising cognitive and emotional components, as outlined by Davis (1980) through the Interpersonal Reactivity Index, which includes perspective-taking, fantasy, empathic concern, and personal distress.

Paradoxically the recent pandemic increased demands for collaborative capacity in communities, even as social distancing was practiced (Bashir et al., 2021; Chauhan et al., 2024). Online and web platform environments, once viewed with suspicion, have gained renewed interest as virtual spaces for collaboration (Bashir et al., 2021). These environments are preferred for their ability to alleviate interpersonal relationship fatigue and enable work and learning without the high expectations of social collaboration (Fung, 2004). However, the online environment has limited effectiveness for activities requiring direct interaction. Despite its limitations, the online environment has become a necessary tool in disaster situations, and we have grown accustomed to its convenience (Hussein et al., 2020). Online collaboration in virtual spaces proved its strength and advantages in connecting people and sharing experiences beyond prolonged disaster situation (Dhawan, 2020; Wang and Sun, 2021).

The limitations and possibilities of the online environment have been extensively explored in the educational context. Online learning has evolved since the 1990s, allowing individuals to take control of their own learning. With the advent of Web 2.0 in the 2000s, web platforms based on sharing networks evolved into collaborative spaces. Technological advances have made online learning activities ambivalent, with individual initiative coexisting with group collaboration (Fung, 2004; Nokes-Malach et al., 2015). The pandemic accelerated the adoption of video conferencing tools like Zoom and collaborative platforms like Slack, creating a more flexible and effective online learning environment for collaboration (Camilleri and Camilleri, 2021).

In terms of online collaboration, it is important to note that the web platform environment requires users to be self-directed, and different levels of cognitive, motivational, and behavioral strength in self-regulation can impact social collaboration (Fung, 2004; DiDonato, 2013). Online collaborative activities can be facilitated when self-regulation is transformed into collaborative regulation, and individuals perceive themselves as effective members of a group with collaborative efficacy. Self-regulation is not a fixed trait, but rather a situational variable that can vary depending on the psychological, physical, and social environment (Bandura, 1989; McCaslin and Hickey, 2001; Zimmerman, 2013). Collaborative regulation explains the transition from individual regulation to group collaboration (Hadwin and Oshige, 2011; DiDonato, 2013).

Disaster researchers believe that a sense of community forms rapidly during a crisis, shaping behaviors of mutual help (Drury et al., 2019). A sense of shared identity is created by shifting from individual to collective consciousness, a process facilitated by collaborative regulation. In learning contexts, individuals can shift their self-regulation to social regulation by adapting their group interactions, thereby creating a sense of collective identity and contributing to cooperative activities with other members (Hadwin and Oshige, 2011; Rogat and Linnenbrink-Garcia, 2011). If one successfully shifts to collective consciousness through collaborative regulation, they can work toward the group’s goals and make the collaboration successful (Volet et al., 2009; Lajoie and Lu, 2012).

Collaborative efficacy, alongside collaborative regulation, facilitates the collaborative process. It refers to an individual’s confidence in their ability to successfully achieve the group’s goals in a collaborative situation (Alavi and McCormick, 2008). Collaborative efficacy is a belief in one’s ability to collaborate (Goddard et al., 2004). Thus, collaborative regulation and efficacy are key mechanisms explaining how individual collaborative tendencies with regulative ability function in disaster or risk situations.

With this background, our study investigates how collaborative characteristics such as collaborative regulation and efficacy, along with self-regulation, interpersonal reactivity, and disaster responses, are related and function together in online collaborative situations constrained by a disaster. We first examined various reactions to the pandemic through a questionnaire and explored the structure of responses by factor analysis. Then, we analyzed the canonical correlation model of individuals’ interpersonal reactivity tendencies, collaborative characteristics, and disaster responses while performing collaborative tasks in a situation where non-face-to-face interaction was inevitable. Finally, we investigated the causal relationships between collaborative regulation, efficacy, and interpersonal reactivity on empathy during the pandemic situation by incorporating mediation and moderation models.

## 2 Methods

### 2.1 Participants

The participants consists of 123 students from a university in a metropolitan area of South, Korea, enrolled in social science course related to disaster issues. This course was offered when the immediate threat and fear from the pandemic had diminished, but the prolonged disaster’s effects, such as social distancing and mask-wearing, still impacted everyday life. The gender distribution is fairly balanced, with 59 female (48.0%) and 64 male (52.0%) participants. All participants were third or fourth-year humanities and social sciences students, with ages ranging from 20 to 24 years. This age group is considered a critical period for the finalization of social dispositions, making them a suitable population for assessing social attributes such as interpersonal reactivity and collaborative characteristics (Caine and Caine, 2015; Andrews et al., 2021). Consequently, purposive convenience sampling was employed as a non-probability sampling method (Etikan et al., 2015).

### 2.2 Procedures

Participants engaged in a non-face-to-face collaborative activity over a seven-week period as part of an online social sciences course focused on risk and disasters. They formed teams and freely chose topics related to various pandemic issues. The activity followed an online learning framework suggested by Han et al. (2014), encompassing stages of team formation, task selection, information sharing, idea generation, and problem-solving. Each team identified and addressed a challenging psychological or social issue related to the pandemic.

A quasi-experimental design was established to ensure the contextuality of the pandemic situation was captured and to explore social interactions in an online environment during a prolonged disaster. To maintain objectivity in collaboration, participants were grouped into teams of 5–6 students with whom they had no prior familiarity, and communication was restricted to online interactions within the web platform learning systems. Responses regarding collaborative characteristics and interpersonal reactivity were collected during the fourth week, after some period of collaborative activity. Data on responses to disaster situations were gathered 1 week following the conclusion of the collaborative activity.

### 2.3 Instrument

Self-regulation was measured as a factor that could impact collaborative traits, along with collaborative regulation and collaborative efficacy, which are involved in group activities. Self-regulation was assessed using a 74-item scale that evaluates the ability to control and utilize cognitive, motivational, and behavioral aspects (Yang, 2000). This measure is considered to have a systematic relationship with collaborative control and can explain multidimensional personal characteristics in collaborative situations (Kim, 2018). Based on DiDonato (2013) theory that self-regulation is converted into collaborative regulation, Lim et al. (2015) developed a six-item measure to assess collaborative regulation. Collaborative efficacy was measured using 21 items from Alavi and McCormick (2008) Self-efficacy for Group Work Measure (2008), which was modified and used by Park (2016). All items in the study were rated on a 5-point scale.

The interpersonal reactivity index (IRI) has been widely used in various fields to measure social reactivity, including empathy, since it was published by Davis in 1980 (Keysers, 2011; Gilet et al., 2013). The IRI consists of 28 items to measure four factors: “Perspective Taking” refers to the tendency to consider and accept the perspective of others, “Fantasy” refers to the degree to which one is immersed in the emotions and situations of characters in movies, plays, and books, “Empathic Concern” refers to the degree to which one sympathizes and feels compassion for others’ situations, and finally “Personal Distress” refers to the degree of discomfort one feels while observing or experiencing distressing situations and suffering (Davis, 1980; Keysers, 2011).

A total of 18 items were presented to describe feelings and perceptions during the disaster situation of the pandemic. By identifying reactions to disaster situations, it is possible to understand how disasters are experienced and perceived, and to understand and explain empathetic and collaborative behaviors in situations (Vardy and Atkinson, 2019). A preliminary study was conducted before the main study, we utilized a sample of similar groups to express a range of thoughts and feelings about the pandemic and extracted common themes to formulate questions. The content of the 18-item questionnaire included items measuring empathy for the situation of vulnerable people such as the elderly, children, and people with medical conditions; fearful reactions to a pandemic; perceptions of being unlucky to experience a disaster; attempts to identify and hold accountable those responsible for the pandemic; and trust in policies and experts. After collecting the participants’ responses, we conducted factor analysis to extract the constructs and check their reliability.

### 2.4 Analysis

Exploratory factor analysis was conducted to extract factors and validate the Disaster Response Measurement Questionnaire. Exploratory factor analysis is advantageous in situations where there are no theoretical factors or when trying to explain the properties of situational data, as it produces statistical values based on given data and reveals the characteristics of the data as factors (Hair et al., 2009; Watkins, 2018). The instrument used to measure reactions to a disaster is explores emotions and perceptions in the context of the situation, making exploratory factor analysis an appropriate method. Principal component analysis and orthogonal varimax rotation were utilized for factor extraction (Watkins, 2018). In determining the number of factors, both the scree plot and Kaiser’s criterion, based on the number of eigenvalues >1, were considered to ensure that the most appropriate number of factors were extracted (Hair et al., 2009). The factors were identified by finding commonalities among the items and were validated by three experts in the fields of disaster, psychology, and measurement.

**Figure 1:**
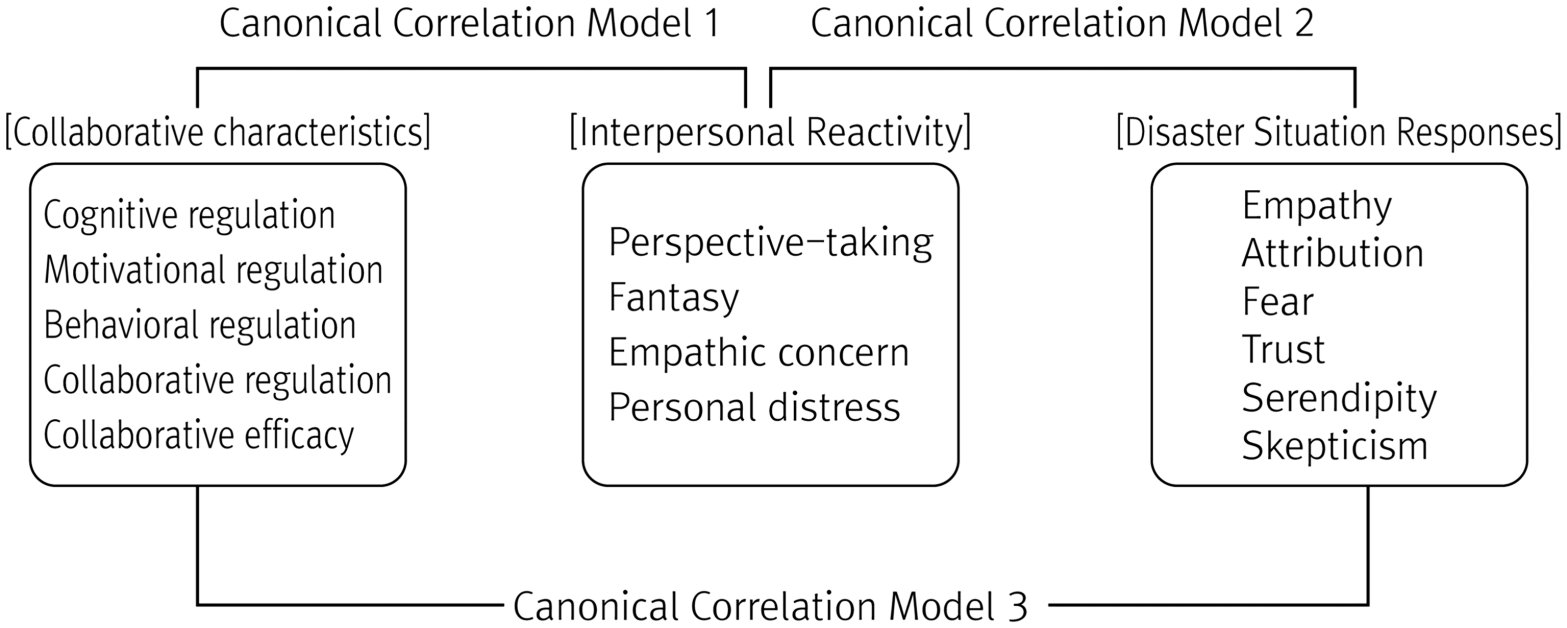
Canonical correlation model.

Canonical correlation analysis was utilized to test the interrelationships between collaborative characteristics, interpersonal reactivity, and disaster responses, which are composed of multiple factors (Sherry and Henson, 2005; Hair et al., 2009). Canonical correlation analysis is a multivariate analysis technique that simultaneously examines the linear relationship between two groups of variables, minimizing the statistical error caused by analyzing the correlation between single variables multiple times (Levine, 1977; Sherry and Henson, 2005; Hair et al., 2009). Three canonical correlation models were constructed to analyze the relationships between the different sets of variables: (1) collaborative characteristics with interpersonal reactivity, (2) interpersonal reactivity with disaster responses, and (3) collaborative characteristics with disaster responses. Canonical correlations were calculated from the combination of variables included in each group of variables. The standardized canonical coefficient was investigated for the significant canonical functions, and the canonical relationship between the variables was interpreted to understand the interrelationships among the variables as depicted in Figure 1.

The PROCESS method (Hayes, 2018) was used to conduct a mediation and moderation effect analysis to explore the causal relationship between collaborative regulation, collaborative efficacy, and interpersonal reactivity to situational empathy among six factors of disaster response in non-face-to-face collaborative situations.

**Figure 2:**
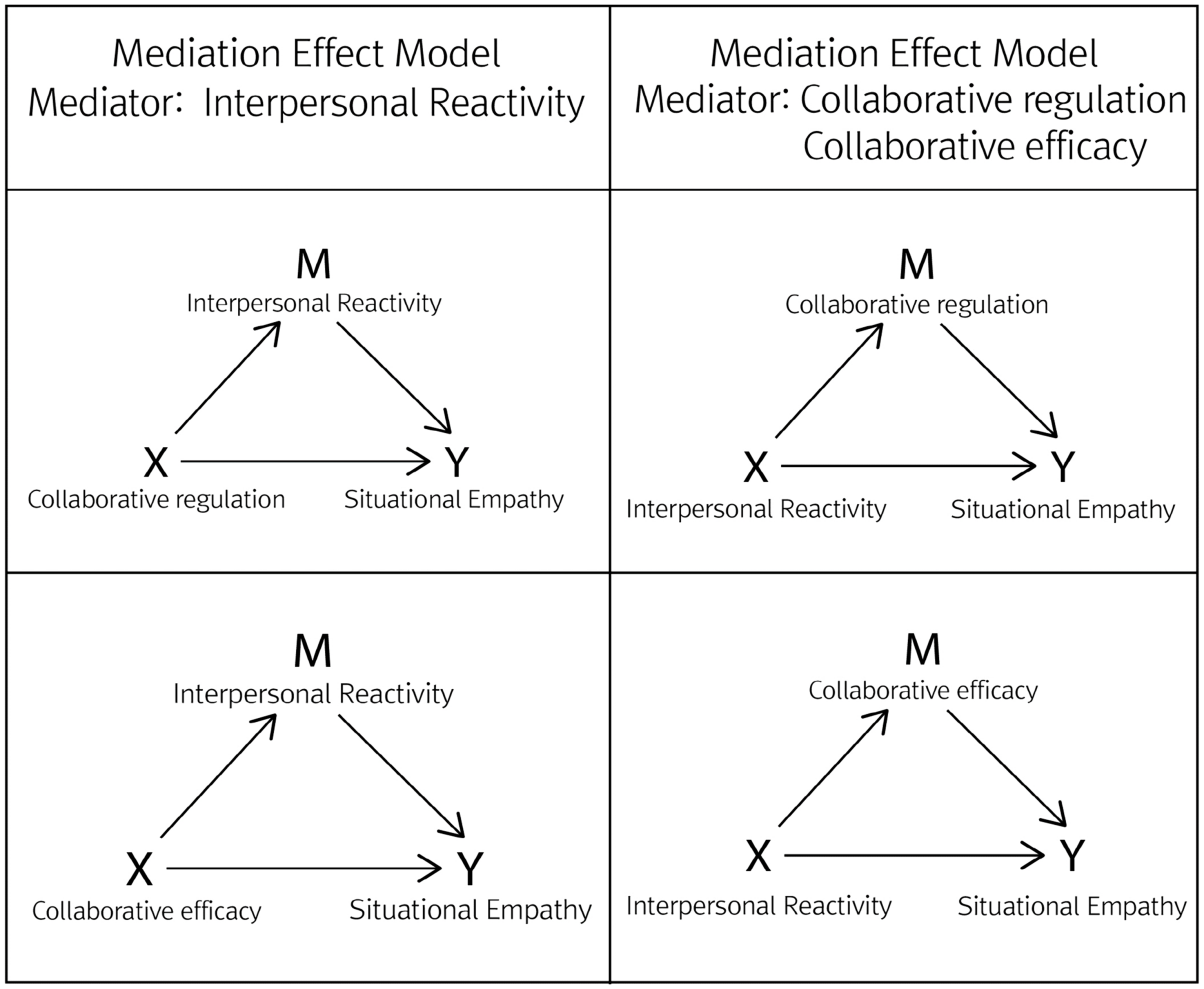
Mediation effect model.

Empathy is considered a critical affective response to disaster and risk situations, serving as an important trigger for altruistic behaviors (Lieberman, 2013; Marsh, 2016). When there is insufficient prior research to establish a model, model exploration can be conducted. In this context, as depicted in Figure 2, a mediator is a mechanism that connects independent and dependent variables, while a moderator is a variable that changes the strength and direction of the relationship (Hayes, 2018).

## 3 Results

### 3.1 Descriptive statistics

Descriptive statistics of the collaborative characteristics, interpersonal reactivity, and disaster situation responses for the 123 subjects are presented in Table 1. The skewness and kurtosis values confirm a normal distribution. Collaborative regulation had the highest mean value (*M* = 4.320, *SD* = 0.651), followed by collaborative efficacy (*M* = 4.214, *SD* = 0.732). While perspective-taking had the highest mean (*M* = 3.773, *SD* = 0.845) in interpersonal reactivity, it was lower than 4.0, suggesting a potential limitation of online interactions. Situational empathy had the highest mean value (*M* = 4.259, *SD* = 0.783) among disaster situation responses, suggesting that empathy remained a strong emotion even in a non-face-to-face situation.

**Table 1:** Descriptive statistics.

Classification	Variables	Mean	SD	Skewness	Kurtosis
Collaborative characteristics	Cognitive regulation	3.565	0.533	0.332	−0.149
	Motivational regulation	3.417	0.569	−0.066	−0.175
	Behavioral regulation	3.259	0.475	0.373	0.503
	Collaborative regulation	4.320	0.538	−0.262	−0.808
	Collaborative efficacy	4.214	0.527	0.183	−0.258
Interpersonal reactivity	Perspective-taking	3.773	0.517	−0.099	−0.024
	Fantasy	3.762	0.732	−0.142	−0.562
	Empathic concern	3.675	0.638	−0.196	−0.582
	Personal distress	3.004	0.729	0.381	−0.130
Disaster situation responses	Empathy (situational)	4.259	0.576	−0.672	−0.041
	Attribution	3.225	0.814	−0.161	−0.391
	Fear	3.877	0.629	−0.336	−0.110
	Trust	3.898	0.578	−0.396	0.473
	Serendipity	3.012	0.823	0.183	0.013
	Skepticism	3.154	0.808	−0.419	−0.084

### 3.2 Factor analysis

Exploratory factor analysis was conducted on a total of 18 items to measure disaster situation responses. The number of components was determined to be between 5 and 7 through the scree plot. Six factors with eigenvalues above 1 were confirmed applying the Kaiser criterion, thus resulting in a six-factor model. The Bartlett’s test of sphericity for the six-factor model of disaster situation responses was significant at 0.001 (*X*^2^ = 773.151, *df* = 153, *p* = 0.000), confirming the adequacy of the factor analysis data and the appropriateness of the model. In addition, the commonality value was >0.50 for all items. The cumulative percentage of variance explained by six factors was 67.590% of total variance.

The factors were named as empathy, attribution, fear, trust, serendipity, and skepticism, and were independently checked by three experts to ensure content validity. The reliability Cronbach’s *α* value was 0.713, indicating adequate reliability to be used in the further analysis (Table 2).

**Table 2:** Factor analysis.

Main concepts of items	Factor loading						Factor naming
	1	2	3	4	5	6	
Feel sad for infected people from disadvantaged or marginalized group	0.842	−0.018	0.112	0.006	−0.038	0.128	Situational empathy
Saddened by the fact that innocent people are infected	0.836	0.052	0.183	−0.077	0.021	0.069	
Feel bad for young children get infected	0.767	0.079	0.152	−0.073	−0.077	0.019	
Give special care to avoid transferring to elderly ones	0.658	−0.073	0.074	0.271	0.214	0.075	
Willing to lend my spare to someone without a mask	0.652	0.027	0.029	0.342	−0.143	−0.149	
Hold specific groups accountable for the spread of this pandemic	0.118	0.824	0.014	−0.009	0.136	−0.013	Attribution
Hold specific countries accountable for their irresponsibility for the outbreak	0.036	0.805	0.021	−0.024	−0.080	0.144	
Hold someone responsible for the economic damage by the pandemic	−0.121	0.804	−0.135	−0.090	0.185	−0.020	
Consider I/my family could be infected with the news of virus spreading	0.161	−0.212	0.845	−0.031	0.008	0.021	Fear
Feel fear by the reports of the virus spreading	0.207	0.047	0.833	0.053	0.166	0.121	
Feel helpless when hearing about the spread of the virus	0.293	0.349	0.491	0.148	0.065	−0.211	
Follow government response policies to prevent the spread of the pandemic	0.073	−0.168	−0.143	0.791	0.040	−0.045	Trust
Practice good personal hygiene to avoid getting virus	0.136	0.054	0.022	0.745	0.078	0.018	
Follow the guidelines of experts in epidemiology to stop the pandemic	−0.070	−0.003	0.265	0.717	−0.219	0.154	
Unlucky ones got virus	−0.082	0.166	0.017	−0.062	0.790	−0.083	Serendipity
Pandemic is an unfortune disaster by the destruction of the nature	0.052	0.015	0.218	0.044	0.711	0.381	
Pandemic is an unavoidable disaster by advances in technology	0.120	−0.015	−0.075	0.094	0.168	0.846	Skepticism
Global failure to prevent of the pandemic	0.018	0.435	0.307	−0.034	−0.127	0.568	

The reliability of the instrument for collaborative characteristics measuring the collaborative regulation, collaborative efficacy, cognitive regulation, motivational regulation, and behavioral regulation was 0.957, and the reliability of the interpersonal reactivity scale was 0.852, which was determined to be sufficient for further analysis.

### 3.3 Canonical correlation analysis

A total of four canonical correlation functions (CCFs) were derived from the canonical correlation analysis between the collaborative characteristics and interpersonal reactivity indexes of the participants. Among four canonical correlation functions, CCF 1 (Wilk’s λ = 0.634, *F* = 3.014) and CCF 2 (Wilk’s λ = 0.804, *F* = 2.345) were statistically significant, as Table 3 presented. This means that collaborative characteristics and interpersonal reactivity are significantly and positively related. The first factor explained 21.3% of the total variance and the second factor explained 14.0%. Examining the standardized correlation coefficients of canonical function 1, reveals that low collaborative efficacy (−0.518) and low perspective taking (−0.730) are related. We can then assume that low motivational regulation (−0.495) is related to low fantasy (−0.355). The standardized canonical coefficients of canonical function 2 indicate that low cognitive regulation (−1.020) is related to low fantasy (−0.876), and high behavioral regulation (1.097) is related to high empathic concern (0.826). These canonical relationships suggest that there is a systematic relationship between collaborative characteristics and interpersonal reactivity, especially the relationship between collaborative efficacy and perspective taking, motivation and cognitive regulation with fantasy, and behavioral regulation and empathic concern.

**Table 3:** Canonical correlation analysis between collaborative characteristics, interpersonal reactivity, and disaster situation responses.

	Model 1		Model 2		Model 3
Group 1: collaborative characteristics	CCF 1	CCF 2	CCF 1	CCF 2	CCF1
Cognitive regulation	−0.262	−1.020			−0.538
Motivational regulation	−0.495	−0.292			−0.445
Behavioral regulation	−0.159	1.097			−0.047
Collaborative regulation	0.214	−0.186			−0.252
Collaborative efficacy	−0.518	0.268			−0.074
Group 2: interpersonal reactivity	CCF 1	CCF 2	CCF 1	CCF 2	
Perspective-taking	−0.730	−0.218	−0.618	0.482	
Fantasy	−0.355	−0.876	0.024	−0.551	
Empathic concern	−0.163	0.826	−0.609	−0.273	
Personal distress	0.459	−0.562	0.249	−0.455	
Group 3: disaster situation responses			CCF 1	CCF 2	CCF 1
Empathy			−0.957	−0.119	−1.012
Attribution			0.158	−0.128	0.077
Fear			−0.017	−0.469	0.003
Trust			−0.037	0.785	0.212
Serendipity			0.159	−0.338	0.155
Skepticism			−0.030	0.101	0.094
*Rc*	0.461	0.374	0.483	0.347	0.530
*Rc^2^*	0.213	0.140	0.233	0.120	0.281
Eigen value	0.269	0.163	0.305	0.137	0.390
*p*-value	0.000	0.007	0.000	0.070	0.000

A total of four canonical functions were derived from the canonical correlation analysis between interpersonal reactivity and disaster situation responses, and canonical function 1 (Wilk’s λ = 0.834, *F* = 2.478) was significant at the level of 0.001, as Table 3 presented. It can be concluded that interpersonal reactivity and disaster situation responses form a significant canonical correlation. The canonical function 2 (Wilk’s λ = 0.828, *F* = 1.608) was significant at the 0.1 level and was included in the analysis process. It was found that the CCF 1 explained 23.3% of the total variation and the CCF 2 explained 12.0%. The standardized canonical coefficients for Canonical Function 1 confirmed that low perspective taking (−0.618) was positively related to low situational empathy (−0.957). Personal distress (0.249) and serendipity about the disaster (0.159) were also suggestive of a positive relationship, although the values of coefficients were relatively small. In canonical function 2, perspective taking (0.482) was associated with trust (0.785) and low fantasy (−0.551) with low fear (−0.469). These canonical relationships indicate that positive interpersonal reactivities, such as perspective taking and fantasy, are associated with positive response like situational empathy and trust. However, negative interpersonal reactivity, such as personal distress, are associated with negative response like feelings of bad luck and fear.

Five canonical functions were derived from the results of the canonical correlation analysis between collaborative characteristics and disaster situation responses. Among them, canonical function 1 (Wilk’s λ = 0.583, *F* = 2.341) was significant at the level of 0.001, as Table 3 presented, confirming the relationship between collaborative characteristics and disaster situation responses. Function 1 explained 28.1% of the total variance. The standardized coefficients of variation for the first model indicated that low cognitive regulation (−0.538) and low situational empathy (−1.012) were positively related. These canonical relationships suggest that individual tendencies of regulation are related to empathy in disaster situations and that cognitive regulation can influence situational empathy.

### 3.4 Mediation and moderation effect analysis

The causal relationships between collaborative characteristics and situational empathy among disaster responses in prolonged disaster situation are further explored by conducting mediation and moderation analyses. For this analysis, collaborative regulation and collaborative efficiency among collaborative characteristics were focused with situational empathy as the main elements of causal relationships, as the variables of highest mean scores (4.270, 4.014, 4.259 in order) in each factor, with interpersonal reactivity.

The PROCESS model 4 was used to analyze the causal effects of collaborative regulation and collaborative efficacy on situational empathy with interpersonal reactivity as mediators. The model for situational empathy was found to be statistically adequate (*R*^2^ = 0.176, *F* = 12.852, *p* < 0.001). By performing bootstrapping techniques for verification, we found that the total effect of collaborative regulation on situational empathy was significant (*t* = 3.756, *p* < 0.001), and the direct effect (*t* = 3.136, *p* = 0.002) and the mediation effect of interpersonal reactivity were significant (95% CI = 0.006, 0.144).

The model for collaborative efficacy was also found to be appropriate at the 0.001 level of significance (*R*^2^ = 0.144, *F* = 20.496, *p* < 0.001). When analyzed by bootstrapping, the total effect (*t* = 4.527, *p* < 0.001) and direct effect (*t* = 4.114, *p* < 0.001) of collaborative efficacy on situational empathy were significant, but the mediation effect of interpersonal reactivity was not significant (95% CI = −0.004, 0.132) (Table 4).

**Table 4:** Mediation effect of interpersonal reactivity index on situational empathy.

Collaborative regulation—Situational empathy	*b*	*se*	*t*	*p*	LLCI	ULCI
Total effect	0.346	0.092	3.756	0.000	0.163	0.528
Direct effect	0.284	0.090	3.136	0.002	0.104	0.464
Indirect effect		Effect	Boot SE	Boot LLCI	Boot ULCI	
Mediator: interpersonal reactivity		0.061	0.035	0.006	0.144	
Collaborative efficacy—Situational empathy	*b*	*se*	*t*	*p*	LLCI	ULCI
Total effect	0.464	0.102	4.527	0.000	0.261	0.667
Direct effect	0.410	0.099	4.114	0.000	0.212	0.607
Indirect effect		Effect	Boot SE	Boot LLCI	Boot ULCI	
Mediator: interpersonal reactivity		0.054	0.034	−0.004	0.132	

The effect of interpersonal reactivity on situational empathy was accessed using PROCESS model 4 to explore the mediation effects of collaborative regulation and collaborative efficacy. The model with collaborative regulation as the mediating effect was statistically significant (*R*^2^ = 0.176, *F* = 12.852, *p* < 0.001). In addition, the total effect (*t* = 3.845, *p* < 0.001) and direct effect (*t* = 3.238, *p* = 0.001) of interpersonal reactivity on situational empathy, and the mediating effect of collaborative regulation were also significant (95% CI = 0.004, 0.186).

The model with collaborative efficacy as a mediator was also found to be appropriate at the 0.001 level of significance (*R*^2^ = 0.219, *F* = 16.833, *p* < 0.001). The total effect (*t* = 3.845, *p* < 0.001) and direct effect (*t* = 3.377, *p* = 0.001) of interpersonal reactivity on situational empathy were significant, but the mediating effect of collaborative efficacy was not significant (95% CI = −0.007, 0.173) (Table 5).

**Table 5:** Mediation effect of collaborative regulation and efficacy on situational empathy.

Interpersonal interactivity—Situational empathy	*b*	*se*	*t*	*p*	LLCI	ULCI
Total effect	0.454	0.118	3.845	0.000	0.220	0.688
Direct effect	0.378	0.116	3.238	0.001	0.146	0.609
Indirect effect		Effect	Boot SE	Boot LLCI	Boot ULCI	
Mediator: collaborative regulation		0.076	0.046	0.004	0.186	
Interpersonal interactivity—Situational empathy	*b*	*se*	*t*	*p*	LLCI	ULCI
Total effect	0.454	0.118	3.845	0.000	0.220	0.688
Direct effect	0.380	0.112	3.377	0.001	0.157	0.603
Indirect effect		Effect	Boot SE	Boot LLCI	Boot ULCI	
Mediator: collaborative efficacy		0.074	0.045	−0.007	0.173	

The moderation effect analysis, after the mediation effect analysis, revealed no moderation effects of interpersonal reactivity on collaborative regulation (95% CI = −1.661, 1.931) and collaborative efficacy (95% CI = −1.818, 2.068), and no moderation effects of collaborative regulation (95% CI = −1.4007, 0.173) and collaborative efficacy (95% CI = −1.542, 1.909) on interpersonal reactivity. This suggests that there are mutual mediation effects of collaborative regulation, interpersonal reactivity, and situational empathy, but no moderation effects of collaborative regulation, collaborative efficacy, or interpersonal reactivity.

The moderation effect model confirmed the direct causal relationship between collaborative regulation, collaborative efficacy, interpersonal reactivity, and situational empathy in a non-face-to-face situation. The PROCESS model analysis method has the advantage of discovering mediation and moderation effects and elaborating the causal relationship between variables, moreover, it can also confirm the direct relationship between variables with mediation effects in cases where moderation effects are absent (Hayes, 2018).

## 4 Discussion

Unpredictable disasters have become more frequent and prevalent, necessitating urgent collaborative responses from communities facing crises. However, disasters often create conditions that challenge individuals’ ability to empathize and collaborate effectively. Paradoxically, the greater the difficulty in collaborating, the more pressing the need for a collective response.

In the early 2020s the world experienced an unprecedented pandemic which presented new challenges. First and foremost, the nature of a pandemic disaster makes natural interaction difficult and necessitates social distancing. While non-face-to-face activities using the Internet have proven effective in prolonged disaster situations where social interaction is limited, we still know little about the psychological mechanisms that enable collaboration, such as how collaborative and social characteristics function in non-face-to-face collaboration situations.

To begin to fill this lacuna, the present study explored and explained relationships among interpersonal reactivity as a social trait inherent in individuals, collaborative characteristics including collaborative regulation and efficacy, and different responses to disaster situations. A canonical correlation model was used to analyze the systematic relationship between interpersonal reactivity, collaborative characteristics, and factors that constitute disaster reactions. Subsequently, mediation and moderation effect models were established to explore the causal relationship between collaborative regulation, collaborative efficacy, and interpersonal reactivity on situational empathy.

The first result of the canonical correlation analysis showed that collaborative characteristics and interpersonal reactivity have a significant and systematic relationship. Specifically, collaborative efficacy with perspective-taking as the willingness to accept others’ positions, motivational and cognitive regulation with fantasy as the imagination of oneself in the shoes of others, and behavioral regulation with empathic concern for others were interrelated. This is particularly notable given that studies have shown that compassion and sympathy for others are the major factors leading to more collaborative and altruistic behavior (Lieberman, 2013; Marsh, 2016).

The analysis for the second canonical correlation model between interpersonal reactivity and disaster responses showed that positive interpersonal reactivity was related to positive disaster responses, and negative interpersonal reactivity was related to negative disaster responses. Specifically, positive interpersonal reactivity that is intrinsic to the individual, such as taking others’ perspectives and imagining oneself in others’ shoes, is associated with positive social responses such as empathizing with the disaster vulnerable and trusting disaster-related policies and experts. However, personal distress, which is the level of discomfort one feels while observing or experiencing a crisis, is associated with negative emotions such as fear and bad luck in disaster situations, even in non-face-to-face collaboration.

In the third canonical correlation model, situational empathy was related to cognitive regulation in disaster response. Considering that cognitive regulation was related to fantasy in the first model of the canonical correlation, we can conclude that an individual’s cognitive regulation is related to their social competence to imagine the emotions and situations of others in non-face-to-face collaborative situations and to feel empathy appropriate to the situation.

Subsequently, the mediation model analysis was conducted and concluded that there is a mutual mediation effect of collaborative regulation and interpersonal reactivity on situational empathy. Therefore, the willingness to take collaborative actions with empathy for disaster victims in crisis situations is obtained through the interrelationship between collaborative regulation and interpersonal reactivity. This study verified that this mutual mediation effect could be influential even through online activities.

On the other hand, no moderation effect was found between collaborative regulation, efficacy, and interpersonal reactivity. If the mediation effect confirms the link between the independent variable and the dependent variable, the moderation effect explains the influence on the strength and direction of the relationship (Hayes, 2018). Therefore, while the mediation effect model confirmed that both collaborative regulation and interpersonal reactivity have direct and indirect effects on situational empathy, the moderation effect model confirmed that collaborative efficacy along with interpersonal reactivity have direct causal effects on situational empathy.

While both are collaborative characteristics, we can assume that collaborative regulation and collaborative efficacy worked differently in this study. Collaborative regulation had a mediated causal relationship with interpersonal reactivity, influencing each other to produce an empathic response. Collaborative efficacy, on the other hand, had a direct relationship with situational empathy without interacting with interpersonal reactivity. This tendency is also verified by the result of canonical correlation analyses, where collaborative efficacy did not show a systematic relationship with interpersonal reactivity.

The results of this study demonstrate how the different reactions to the prolonged disaster in the non-face-to-face collaboration context are related to, and work in conjunction with, social characteristics such as interpersonal reactivity that is intrinsic to the individual. It also confirms that the collaborative characteristics exhibited in the non-face-to-face collaboration context are related to interpersonal reactions and perceptions of the disaster situation. The range of responses that individuals feel and perceive in the face of a disaster may work in a complicated way and include not only positive emotions such as empathy and trust but also perceptions and feelings of blame, fear, skepticism, and serendipity. As Brown (2021) explains, a single situation can evoke a wide range of emotions and reactions.

The current generation has experienced global and prolonged disasters and faces an increasing series of social and economic challenges. Disasters can happen so suddenly that those facing them may feel anxious and fearful in the face of uncertainty and find it difficult to respond rationally and reasonably. However, recent research suggests that people empathize with others who feel anxious and fearful of disasters and take collaborative action, which increases the community’s ability to respond (Drury et al., 2019). Even in a non-face-to-face environment, people can be collaborative by drawing on their own embedded social characteristics to shape their perceptions and emotions in response to a disaster. For future research, it is essential to design methodologies that can effectively capture potential variations in perception and emotion during such events. Implementing a before-and-after measurement or a time-series design would be beneficial, as it allows for the assessment of changes over time, while controlling for external social interactions to achieve a more rigorous experimental design. Additionally, considering the initial status of perception at the study’s outset will provide a more comprehensive understanding of how perceptions evolve.

This study also verified that having positive experiences and being responsive in relationships with others are linked to positive responses in disaster situations. People who maintain positive interpersonal relationships and are responsive in their communities may be more inclined to help and collaborate with others in an actual disaster. Therefore, we can say that helping disaster victims to maintain positive emotions in the face of a disaster can foster empathy and subsequent collaboration.

The experience of having to work collaboratively in a non-face-to-face environment in the wake of a disaster deserves more research and attention (Vardy and Atkinson, 2019). Understanding how and to what extent empathy and collaboration are experienced in the aftermath of a disaster, as learned from and in subsequent crises, depends on the content and interpretation of previous experiences.

The interpretation of the results is constrained by the study’s focus on participants in their early 20s, selected for their homogeneity in social brain development. Given the quasi-experimental nature of the study, selection bias and contextual factors could impact the findings and limit their interpretation. To enhance the generalizability of the findings, future research should include a broader range of age groups across various spatial–temporal contexts. This approach would provide a more comprehensive understanding of online collaborative behavior in prolonged disaster situations.

## Data Availability

The raw data supporting the conclusions of this article will be made available by the authors, without undue reservation.
